# A modified method to treat severe asymptomatic pre‐existing degeneration of adjacent segment: a retrospective case‐control study

**DOI:** 10.1186/s12893-021-01163-w

**Published:** 2021-03-23

**Authors:** Xinliang Zhang, Jinwen Zhu, Yibing Li, Dingjun Hao, Wenjie Gao

**Affiliations:** Department of Spine Surgery, Xi’an Honghui Hospital, Xi’an Jiaotong University, Xi’an, Shaanxi China

**Keywords:** Posterior lumbar interbody fusion, Adjacent segment degeneration, Asymptomatic pre‐existing degeneration

## Abstract

**Background:**

Pre-existing degeneration of adjacent segment is an important risk factor for adjacent-segment degeneration (ASD), but only limited and controversial studies have addressed its management.

**Methods:**

We retrospectively analyzed patients with symptomatic degeneration of the L5/S1 segment warranting surgical interference and severe asymptomatic degeneration of the L4/5 segment. Of these patients, those who underwent interbody fusion of the causative (L5/S1) segment and distraction of the intervertebral space and facet fusion of the adjacent L4/5 segment were included in Group A (n = 103), while those who underwent interbody fusion of both the L5/S1 and L4/5 segments were included in Group B (n = 81). Clinical and radiographic outcomes were evaluated.

**Results:**

Mean follow-up time was 58.5 months (range, 48–75 months). We found no significant difference in clinical outcomes or incidence of ASD in the L3/4 segment between Groups A and B. Compared with Group B, Group A experienced less bleeding (315 ± 84 ml vs. 532 ± 105 ml), shorter operation time (107 ± 34 min vs. 158 ± 55 min) and lower costs (US $13,830 ± $2640 vs. US $16,020 ± $3380; *P <* 0.05). In Group A, the disc height ratio (DHR) of the L4/5 segment was significantly increased from a preoperative value of 0.40 ± 0.13 to a last–follow-up value of 0.53 ± 0.18 (*P <* 0.05), while the degree of canal stenosis (DCS) was decreased from a preoperative value of 34.3 ± 11.2% to a last–follow-up value of 15.9 ± 9.3 % (*P <* 0.05).

**Conclusions:**

This modified method could be effective in treating severe asymptomatic pre-existing degeneration of adjacent segment in the lumbar spine.

**Supplementary Information:**

The online version contains supplementary material available at 10.1186/s12893-021-01163-w.

## Background

Posterior lumbar interbody fusion (PLIF), which can provide posterior fusion concomitant with anterior column support, is an effective procedure for lumbar degenerative diseases such as lumbar disc herniation, degenerative spondylolisthesis, degenerative instability and spinal canal stenosis. However, many complications have emerged with its wide application, including pseudarthrosis, loosening or breaking of the implant and adjacent segment degeneration (ASD). Development of ASD has attracted increasing attention because it adversely affects long-term clinical outcome and sometimes requires surgery [1–3]. The exact cause of ASD remains controversial, yet some risk factors have been documented [4, 5], one of the most important being pre-existing degeneration of the adjacent segment [1, 4, 6]. Studies concerning whether asymptomatic pre-existing degeneration of the adjacent disc should be treated, and how it should be treated, are limited and controversial. If it is left untreated, clinical symptoms may appear, warranting a second operation, which is consistently associated with technical difficulties, poor outcomes and psychological and economical burdens [6, 7]. A better therapeutic approach should be taken during the first surgery. Some surgeons might perform a fusion of the causative segment and simultaneous extensive laminal or semi-laminal decompression surgery, or they might augment the vertebral canal in a hidden-proceed manner without fusion of the adjacent segment that has asymptomatic pre-existing degeneration. However, these approaches might not be as effective and can even induce symptomatic ASD [4]. Some researchers suggest a complete PLIF at the adjacent level, which can increase not only cost but also surgical trauma [8]. Recently, some other surgeons have expressed a preference for dynamic stabilization, but multiple studies show that this method offers no significant advantages in preventing ASD and that it poses many issues such as limited indications, dissatisfactory long-term results and high incidence of reoperation [9–11].

In this paper, we report a modified method for treating severe asymptomatic pre-existing degeneration of adjacent segment: performing a distraction of the intervertebral space and facet fusions of the adjacent segment that shows significant degeneration. To evaluate the effectiveness of this approach, we conducted a retrospective case-control study.

## Methods

### Patient characteristics

This study was approved by the Ethics Committee of Honghui Hospital, Xi’an, China (No. XAHHLLSP0901), and written consent was obtained from all patients. We retrospectively reviewed the records of all patients who underwent lumbar spinal fusion surgery of the L4/5 and L5/S1 segments for lumbar disc herniation from September 2009 through November 2014. Cases were consecutive and reflected each surgeon’s surgical preference. We included those who had undergone imaging examinations and preoperative nerve electrophysiological tests to confirm that the S1 nerve root was causative of the neurological symptoms and that the L5/S1 segment was the causative segment. If diagnosis could not be confirmed in the abovementioned examinations, nerve root block had been conducted.

### Inclusion and exclusion criteria

Inclusion criteria were as follows: (1) symptomatic degeneration of the L5/S1 segment warranting surgical interference; and (2) asymptomatic degeneration of the L4/5 segment with Pfirrmann Grade IV [12] or higher causing ≥ 25 % spinal canal stenosis (degree of canal stenosis [DCS] was evaluated and calculated based on preoperative magnetic-resonance imaging [MRI] results per the method introduced by Imagama et al. [13], as shown in Fig. 2b).

**Fig. 1:**
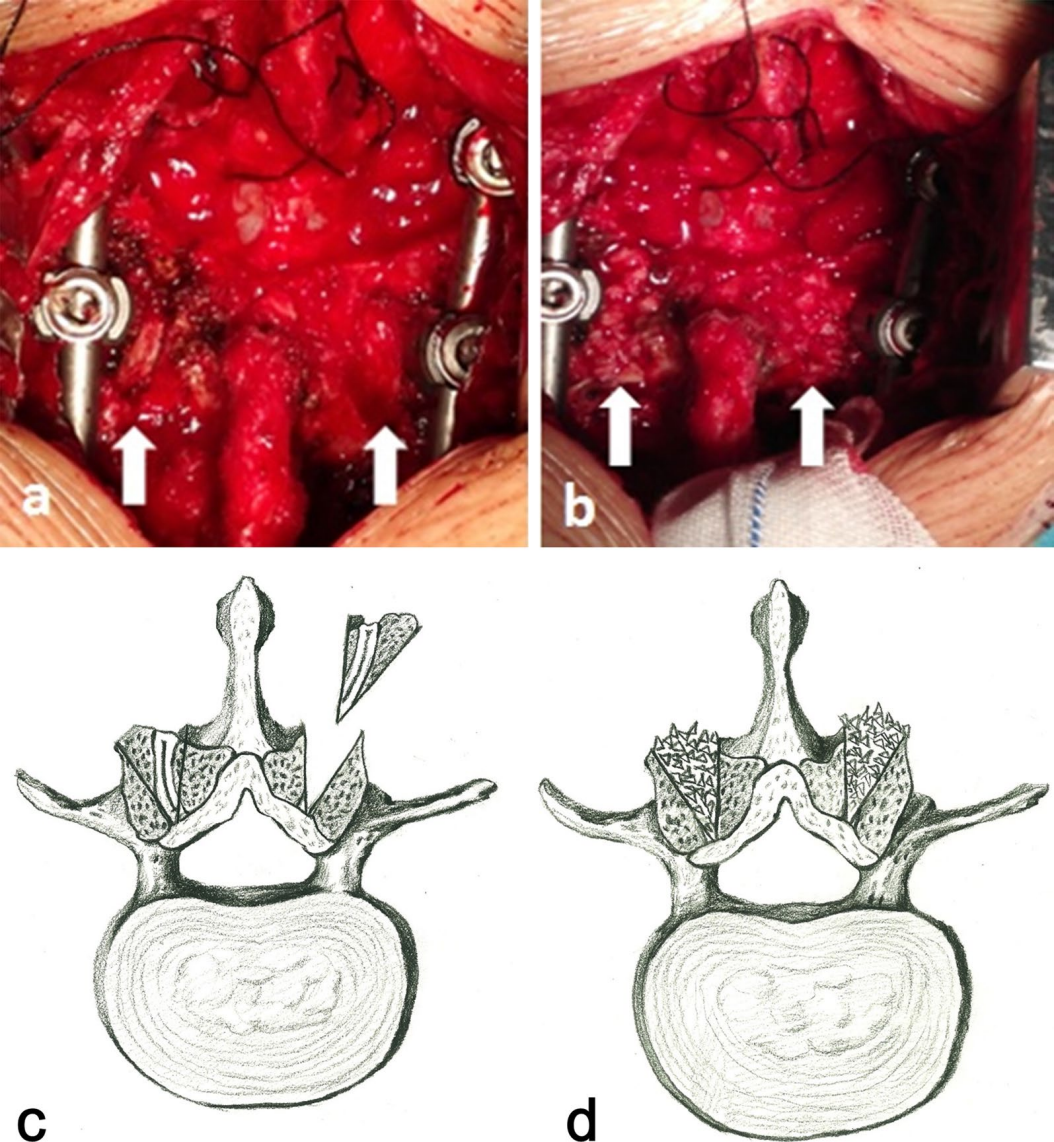
A V-shaped osteotomy was performed on the L4 inferior articular facet and L5 superior articular facet from top to bottom, using an osteotome to create a V-shaped groove on either side of the L4/5 segment (**a, c**). The autogenous bone chips from the laminectomy were then implanted into these grooves (**b, d**)

Exclusion criteria were as follows: (1) symptomatic disc herniation of the L4/5 segment; (2) Pfirrmann Grade IV or higher disc degeneration, spondylolisthesis, spinal instability or stenosis in the L3/4 segment; and (3) history of spinal operation.

Patients who underwent interbody fusion of the causative (L5/S1) segment, as well as a distraction of the intervertebral space and facet fusions of the adjacent L4/5 segment, were included in Group A. Those who underwent interbody fusions of both the L5/S1 and L4/5 segments were included in Group B.

### Surgical technique

For patients in Group A, pedicle screws at L4, L5 and S1 were placed, and standard PLIF of the L5/S1 segment was performed. To distract the intervertebral space in the L4/5 segment, we moderately distracted the disc space between L4 and L5 using a distraction device, using the L4 and L5 pedicle screw caps as fulcrums (Additional file 1: Fig. S1). As a reference for distraction distance, the height of the adjacent intervertebral space was used. We avoided excessive distraction to prevent excessive nerve root tension. For facet fusions of the L4/5 segment, we performed a V-shaped osteotomy on the L4 inferior articular facet and L5 superior articular facet from top to bottom, using an osteotome to create a V-shaped groove on either side of the L4/5 segment (Fig. 1a and c). The autogenous bone chips from the laminectomy were then implanted into these grooves (Fig. 1b and d).

**Fig. 2:**
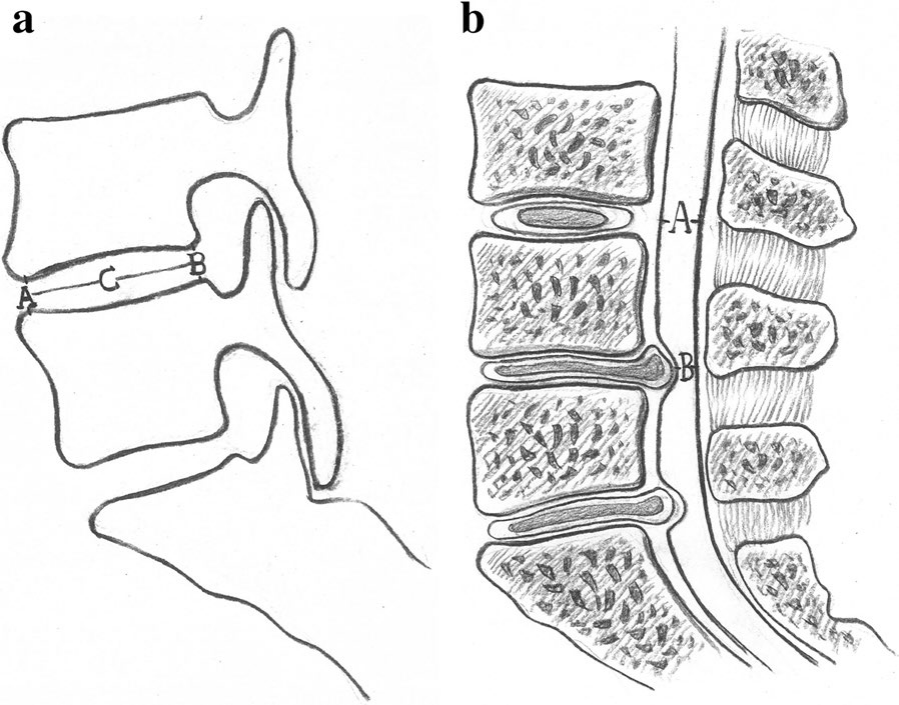
Schematic drawings of calculations of disc height ratio (DHR) and degree of canal stenosis (DCS). **a** DHR = (A + B) / C; **b** DCS = (A − B) / A × 100

For patients in Group B, pedicle screws at L4, L5 and S1 were placed and standard PLIFs of both the L5/S1 and L4/5 segments were performed.

## Assessment of outcome

### Data collection and analysis

X-rays were obtained at 3, 6, 9, 12, 18 and 24 months and every 1 year thereafter to evaluate the position of the internal fixation, presence of loosening or migration and change in the adjacent L3/4 segment. We evaluated symptomatic and functional recovery using the Visual Analogue Scale (VAS) and the Oswestry Disability Index (ODI). At the most recent follow-up, we acquired images via X-ray, computed tomography (CT) and MRI.

We measured the disc height ratio (DHR) of the L4/5 segment per Farfan’s method [14] on lateral radiograph, as shown in Fig. 2a. Briefly, DHR was defined as follows:

$$\frac{{{\text{Anterior disc height }}\left( {\text{A}} \right){\text{ }} + {\text{ posterior disc height }}({\text{B}})}}{{{\text{Distance between the anterior and posterior margins of the disc }}\left( {\text{C}} \right)}}$$

or

$${\text{DHR}} = {\text{ }}({\text{A}} + {\text{B}})/{\text{C}}$$

The degree of disc degeneration in the L3/4 and L4/5 segments was graded on MRI T2-weighted images according to the Pfirrmann grading system [12]. We evaluated and calculated DCS in these segments on MRI per the method introduced by Imagama et al. [13], as shown in Fig. 2b. Briefly, DCS was defined as follows:

$$\frac{{{\text{Sagittal length of the spinal canal }}\left( {\text{A}} \right) - {\text{maximal sagittal length of hernia }}({\text{B}})}}{{{\text{Sagittal length of the spinal canal }}\left( {\text{A}} \right){\text{ }} \times {\text{1}}00}}$$

or

$${\text{DCS}} = {\text{ }}({\text{A}} - {\text{B}})/{\text{A}} \times 100$$

Two observers independently took measurements twice; both interobserver and intraobserver reliability of these data were greater than a correlation of 0.80. Radiographic ASD in the L3/4 segment was defined as follows: (1) development of spondylolisthesis or spinal instability; and (2) Pfirrmann Grade IV or higher disc degeneration.

### Statistical analysis

We compared preoperative with postoperative values using paired *t* tests. An independent sample *t* test was conducted to compare the outcomes between Groups A and B. We conducted statistical analyses using SPSS version 20.0 (IBM Corp., Armonk, NY, USA). Two-sided *P <* 0.05 was considered statistically significant.

## Results

### Follow‐up of patient characteristics

A total of 103 patients (43 male, 60 female) with a mean age of 61.5 years (range, 31–74 years) were included in Group (A) Meanwhile, a total of 81 patients (37 male, 44 female) with a mean age of 59.7 years (range, 34–72 years) were included in Group (B) Mean follow-up time was 58.5 months (range, 48–75 months).

### Clinical results

As shown in Table 1, compared with Group B, Group A experienced less bleeding (315 ± 84 mL vs. 532 ± 105 ml), shorter operation time (107 ± 34 min vs. 158 ± 55 min) and lower costs (US $13,830 ± 2640 vs. US $16,020 ± 3380; all *P <* 0.05).

**Table 1:** Clinical outcomes in Groups A and B

	Group A	Group B
Bleeding (ml)^#^	315 ± 84	532 ± 105
Operation time (min)^#^	107 ± 34	158 ± 55
Total cost (US$)^#^	13,830 ± 2640	16,020 ± 3380
VAS*		
Preoperative	7.7 ± 2.1	7.5 ± 1.6
Final Follow-Up	1.4 ± 0.7	1.6 ± 0.8
ODI*		
Preoperative	65.3 ± 11.6	62.2 ± 8.2
Final Follow-Up	13.5 ± 5.7	14.7 ± 7.3

^#^*P* < 0.05 between Groups A and B

*P < 0.05 between preoperative score and final follow-up score in both Groups A and B

VAS, Visual Analog Scale; ODI, Oswestry Disability Index

Preoperative and final follow-up VAS and ODI scores are presented in Table 1. Preoperatively, the mean VAS score was 7.7 ± 2.1 for patients in Group A and 7.5 ± 1.6 in Group B (*P >* 0.05), and the mean ODI score was 65.3 ± 11.6 and 62.2 ± 8.2 for patients in Group A and 7.5 ± 1.6 in Group B (*P >* 0.05). And Clinical symptoms in both groups were improved postoperatively (*P <* 0.05), but there was no significant difference in postoperative VAS or ODI score between the two groups (all *P* > 0.05).

As for complications, in Group A, one patient experienced a dural injury intra-operatively, which was repaired without severe complication, and one patient experienced a superficial wound infection on postoperative day 6 and was treated with intravenous and later oral antibiotics. In Group B, one patient developed deep post-operative wound infection treated with an incision and debridement followed by intravenous antibiotics. No implant loosening or breakage was found in any patient at the last follow-up.

### Radiographic findings

In Group A, no collapse on the disc height of the L4/5 segment has been found at the last follow-up, and the DHR of the L4/5 segment was significantly increased from a preoperative value of 0.40 ± 0.13 to a last–follow-up value of 0.53 ± 0.18 (*P <* 0.05), while DCS was decreased from a preoperative value of 34.3 ± 11.2 % to a last–follow-up value of 15.9 ± 9.3 % (*P <* 0.05; Table 2). In Group B, the DHR of the L4/5 segment was significantly increased from a preoperative value of 0.39 ± 0.15 to a last–follow-up value of 0.51 ± 0.16 (*P* < 0.05). And there was no significant difference in both pre- and postoperative DHR values between the two groups. Radiographic ASD in the L3/4 segment was found in 13 of 103 (12.62%) patients in Group A and 9 of 81 (11.11%) patients in Group B at final follow-up, but the two groups did not differ significantly in the occurrence of radiographic L3/4 ASD (*P* > 0.05). No symptomatic ASD was found in either Group A or Group B.

**Table 2:** Radiologic Outcomes of L4/5 Segment in Group A

	Preoperative	Final follow-up
DHR^#^	0.40 ± 0.13	0.53 ± 0.18
DCS^#^	34.3 ± 11.2%	15.9 ± 9.3%

^#^*P* < 0.05 between preoperative score and final follow-up score

DHR, Disc Height Ratio

DCS,Degree of Canal Stenosis

### Illustrative case

**Fig. 3:**
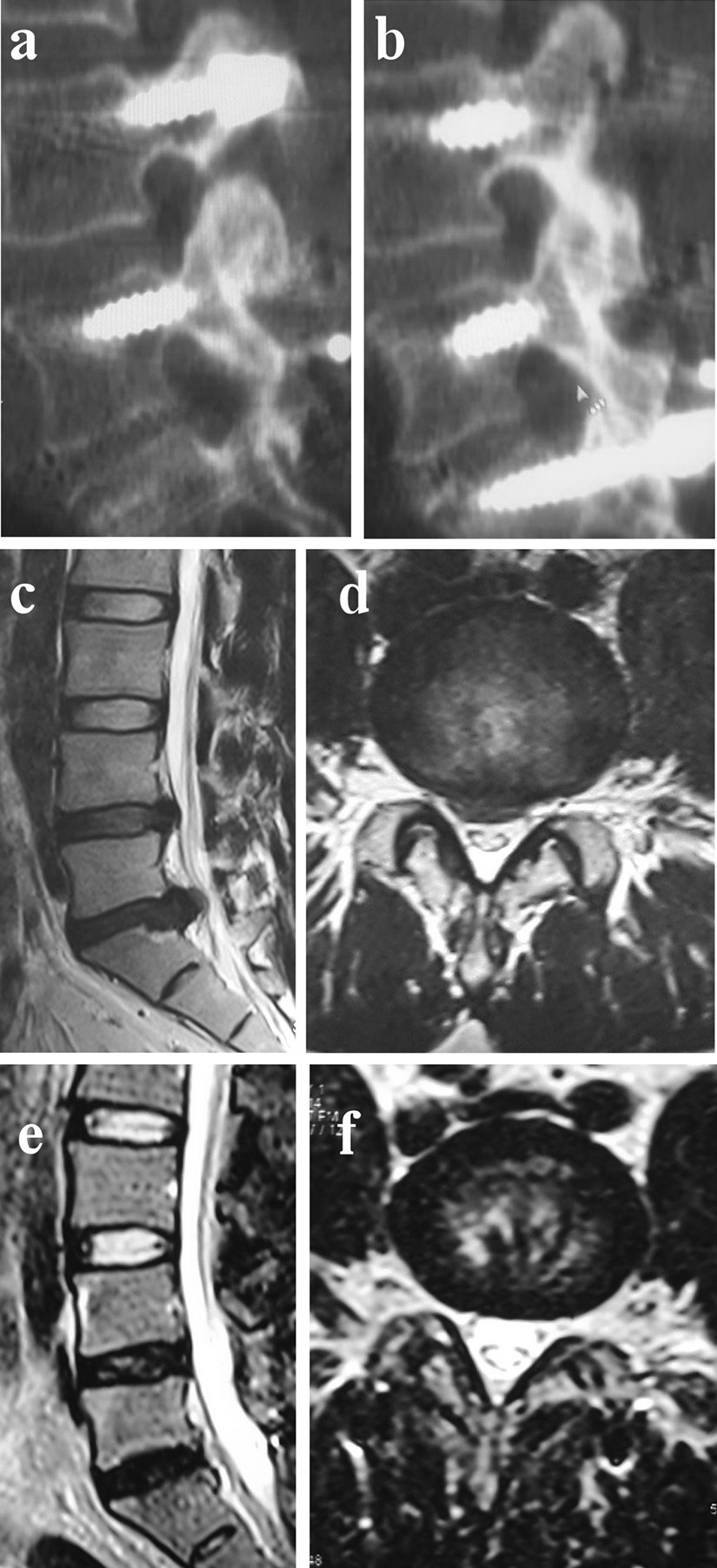
A 54-year-old patient from Group A was followed up for 5 years. Preoperative MRI demonstrated severe disc degeneration in the L4/5 segment (**c**,** d**). We performed standard PLIF of the L5/S1 segment (the causative segment), as well as a distraction of the intervertebral space and facet fusions of the L4/5 segment. At the final follow-up, CT showed that complete fusion of the L4-5 facets had been achieved: left-side facet (**a**); right-side facet (**b**). MRI demonstrated that DCS in the L4/5 segment had decreased (**e**,** f**)

A 54-year-old man with a chief complaint of radiating pain to the left lower extremity was diagnosed as lumbar disc herniation. The causative segment had been confirmed to be the L5/S1 segment by preoperative examination. Preoperative MRI showed that 42.5 % of spinal canal stenosis in the L4/5 segment was caused by the degenerative disc (Pfirrmann Grade IV; Fig. 3c–d). We performed a standard PLIF of the L5/S1 segment and distraction of the intervertebral space and facet fusion of the L4/5 segment. At the 5-year follow-up assessment, CT showed that complete facet fusion of the L4-5 facets had been achieved (Fig. 3a–b), and MRI showed that the degree of spinal canal stenosis in the L4/5 segment had decreased to 7.2 % (Fig. 3e–f), indicating that the disc herniation was retracted.

## Discussion

Our study demonstrated that a distraction of the intervertebral space and facet fusions of severely degenerated L4/5 segment could be effective in preventing ASD of such segments; and that this method had several advantages over interbody fusions of both the L5/S1 and L4/5 segments, including decreased bleeding, shorter operation time and lower costs.

The incidence of ASD varies from 8.2 to 18.5% among studies; about 2.7–20 % of patients with symptomatic ASD need surgical intervention [1–3, 5]. Patients with pre-existing degenerative changes in an adjacent motion segment have worse long-term clinical outcomes and experience higher rates of sequelae [1, 4, 6]. Anandjiwala et al. found that adjacent segments with Pfirrmann Grade IV or V degeneration constitute a significant risk factor for ASD and suggested that these segments should be included in the primary fusion procedure to prevent subsequent ASD [15]. However, complete interbody fusion at the adjacent level not only markedly increases overall operation costs but also leads to more surgical trauma, with related consequences [8]. Some surgeons choose to augment the vertebral canal in a hidden-proceed manner without fusion of the adjacent segment, but Hikata et al. reported that simultaneous decompression surgery without fusion might not be effective in reducing the incidence of symptomatic ASD [4].

Interspinous distraction devices and dynamic stabilization across pedicle screws can theoretically prevent ASD by preserving some motion at the segment. Korovessis et al. recommended the use of the Wallis interspinous implant (Abbott Spine, Bordeaux, France) in the unfused vertebral segment cephalad to instrumentation with mild arthritic changes to protect adjacent segments [16]. In the current study, significant disc degeneration and spinal stenosis in the adjacent segment (L4/5) were present; therefore, distracting the interspinous process is far from enough to retract the herniated disc and decompress the spinal stenosis. The Graf ligament and the Dynesys Dynamic Stabilization System (Zimmer Inc., Warsaw, IN, USA) are the most commonly used devices for dynamic stabilization. Since our patients had severe disc degeneration at the L4/5 segment, the Graf ligament was unsuitable for them [17]. The Dynesys system can resist both tensile and compressive forces and control motion of the segment in all directions, but it has a high incidence of screws loosening [18], and incidence of ASD with this method is similar to that accompanying the traditional method [10, 19].

The current study had several limitations. It was a retrospective study that limited data to what was available in the patients’ medical charts. Only a single center was involved, and a small sample size of cases was included. Patients with significant L3/4 degeneration were excluded to lower interindividual and intergroup variability, but this could have resulted in lower generalizability. Additional prospective studies are necessary to confirm these results.

## Conclusions

Our outcomes indicated that distraction of the intervertebral space and facet fusions could be effective in treating severe asymptomatic pre-existing degeneration of the adjacent segment in the lumbar spine.

## Supplementary Information

**Additional file 1.** To distract the intervertebral space in the L4/5 segment, we moderately distracted the disc space between L4 and L5 using a distraction device, using the L4 and L5 pedicle screw caps as fulcrums.

## Data Availability

The datasets used and/or analyzed during the current study are available from the corresponding author on reasonable request.

## Abbreviations

ASD: Adjacent-segment degeneration
PLIF: Posterior lumbar interbody fusion
VAS: Visual Analogue Scale
ODI: Oswestry Disability Index
MRI: Magnetic resonance imaging
CT: Computed tomography
DHR: Disc height ratio
DCS: Degree of canal stenosis
