# Supplementary figures and images for: A modified method to treat severe asymptomatic pre‐existing degeneration of adjacent segment: a retrospective case‐control study
Source: BMC Surg. 2021 Mar 23;21:161. doi: 10.1186/s12893-021-01163-w (PMC7989102; doi:10.1186/s12893-021-01163-w)

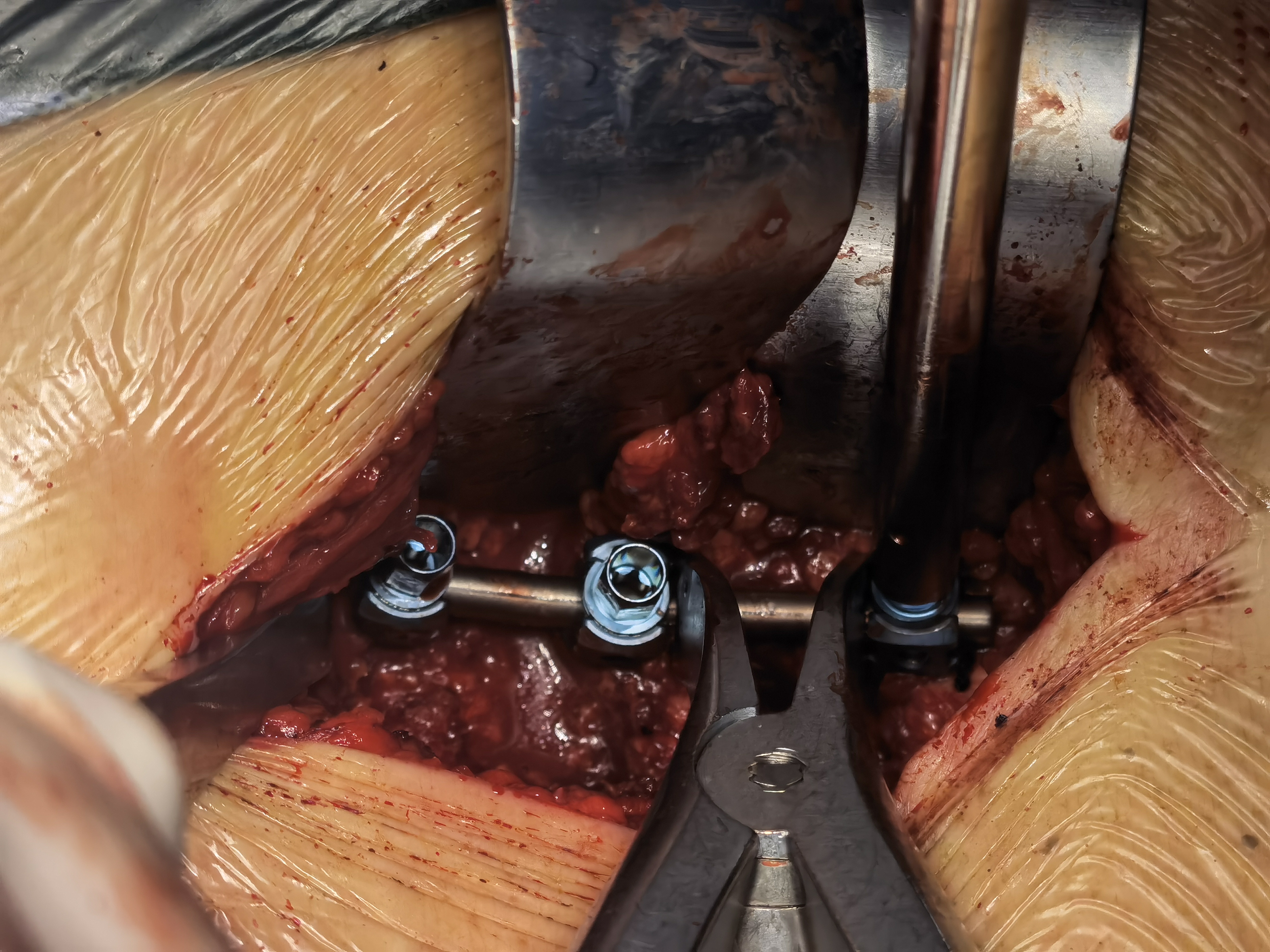

Supplement: Supplementary file 1 — Additional file 1. To distract the intervertebral space in the L4/5 segment, we moderately distracted the disc space between L4 and L5 using a distraction device, using the L4 and L5 pedicle screw caps as fulcrums. [file 12893_2021_1163_MOESM1_ESM.jpg]
